# Immunomodulatory Role of NK Cells during Antiviral Antibody Therapy

**DOI:** 10.3390/vaccines9020137

**Published:** 2021-02-08

**Authors:** Mar Naranjo-Gomez, Marine Cahen, Jennifer Lambour, Myriam Boyer-Clavel, Mireia Pelegrin

**Affiliations:** 1 IGMM, Univ Montpellier, CNRS, Montpellier, France; maria.naranjo@inserm.fr (M.N.-G.); marine.cahen@unilim.fr (M.C.); jenniferlambour@gmail.com (J.L.); 2 Montpellier Ressources Imagerie, Biocampus, Univ Montpellier, CNRS, Montpellier, France; myriam.boyer-clavel@igmm.cnrs.fr

**Keywords:** antiviral immunity, antiviral monoclonal antibodies, immunotherapy, NK cells, inhibitory receptors, B-cell helper neutrophils, vaccine-like effects, CD39, PD-1, PD-L1

## Abstract

Monoclonal antibodies (mAbs) are now considered as a therapeutic approach to prevent and treat severe viral infections. Using a mouse retroviral model, we showed that mAbs induce protective immunity (vaccinal effects). Here, we investigated the role of natural killer (NK) cells on this effect. NK cells are effector cells that are crucial to control viral propagation upon mAb treatment. However, their immunomodulatory activity during antiviral mAb immunotherapies has been little studied. Our data reveal that the mAb treatment of infected mice preserves the functional activation of NK cells. Importantly, functional NK cells play an essential role in preventing immune dysfunction and inducing antiviral protective immunity upon mAb therapy. Thus, NK cell depletion in mAb-treated, viral-infected mice leads to the upregulation of molecules involved in immunosuppressive pathways (i.e., PD-1, PD-L1 and CD39) on dendritic cells and T cells. NK cell depletion also abrogates the vaccinal effects induced by mAb therapy. Our data also reveal a role for IFNγ-producing NK cells in the enhancement of the B-cell responses through the potentiation of the B-cell helper properties of neutrophils. These findings suggest that preserved NK cell functions and counts might be required for achieving mAb-induced protective immunity. They open new prospects for improving antiviral immunotherapies.

## 1. Introduction

Neutralizing monoclonal antibodies (mAbs) are now considered as promising, high added-value therapeutic agents for the prevention and treatment of severe viral infections, including newly emerging viral infections [1,2,3]. The high therapeutic potential of antiviral mAbs is due to their multiple mechanisms of action [4,5]. Thus, through their Fab (fragment of antigen binding) region, they are able to neutralize virions via the specific recognition of viral determinants required for receptor binding and/or entry into host cells. Such direct recognition of viral surface antigens is also able to inhibit the cell–cell transmission of virions. Antibody biological activity is also mediated by the Fc (fragment crystallizable) moiety that displays a variety of effector functions, including binding to complement and Fc receptors (FcRs) expressed by many cells of the immune system. Therefore, upon the recognition of their target antigens, antiviral mAbs can mediate the virus and infected cells elimination via multiple Fc-mediated mechanisms such as complement-dependent cytotoxicity (CDC), antibody-dependent cell-mediated cytotoxicity (ADCC) and antibody-dependent cellular phagocytosis (ADCP) [4,5]. In addition to playing a key role in viral blunting, the mAb therapeutic effect can also be mediated via the induction of protective immunity (vaccinal effect) (reviewed in [6]) which occurs in an Fc-dependent manner [7,8,9,10]. Along these lines, we, and others, have shown enhanced humoral and cellular antiviral immune responses upon antiviral antibody therapy in several preclinical models of viral infection [6,10,11,12,13,14,15]. Importantly, such vaccinal effects have recently been reported in clinical trials involving anti-HIV broadly neutralizing mAbs [16,17,18]. However, the main cellular and molecular mechanisms at play remain ill-understood. A better understanding of antibody-mediated immunomodulation mechanisms is therefore needed to maximize the therapeutic effect of antiviral mAbs.

Preclinical models of viral infection are valuable tools to identify the main mechanisms involved in the induction of vaccinal effects by mAbs. Among them, the infection of immunocompetent mice with the Murine Leukemia Virus FrCasE permits an extensive and integrative analysis of antiviral immune responses under the conditions of both chronic infection and disease development. Using this retroviral infection model, we previously showed that the induction of protective immunity by antiviral mAb requires Fc–FcγRs interactions and involves different FcγR-expressing cells such as dendritic cells (DCs) and neutrophils. In particular, the therapeutic mAb forms immune complexes (ICs) with viral determinants (i.e., virions and/or infected cells) which bind to FcγRs expressed on DCs. IC–FcγRs interaction results in enhanced DC activation and the induction of protective virus-specific CD8^+^ T-cell responses [8]. MAb treatment also induces potent humoral responses which rely on the immunomodulatory functions of neutrophils upon FcγR-triggering. Notably, activated neutrophils acquire B-cell helper functions (i.e., the production of the B-cell activating factor (BAFF)) and induce protective humoral immunity [19]. These observations highlight that multiple FcγR-expressing cells are involved and cooperate to induce vaccinal effects upon mAb therapy. Here, we investigated the role of natural killer (NK) cells in mAb-mediated immunomodulation in infected mice. NK cells are FcγR-expressing innate effector cells that play a key role in the control of viral propagation upon mAb therapy through the killing of infected cells via ADCC [20,21,22]. In addition to their cytotoxic activity, NK cells display multiple immunomodulatory functions. Thus, NK cells can secrete multiple chemokines (i.e., CCL3, CCL4, CCL5, …) and cytokines (IFNγ, TNFα, GM-CSF, …) able to recruit and activate several cells of the immune system [23,24,25,26,27]. NK cell-secreted pro-inflammatory cytokines are involved in the activation/maturation of DCs, the enhancement of T-cell responses as well as in the recruitment and survival of neutrophils [27,28,29,30,31,32]. This has led in recent years to a growing interest in exploiting the immunomodulatory function of NK cells in immunotherapy approaches for cancer and viral infections [33,34,35,36]. In addition, recent studies have reported that NK cells can also play regulatory functions leading to immunosuppressive responses [27,37,38]. Furthermore, it has also been shown that inflammatory conditions can sensitize and activate NK cells in a context-dependent manner [27]. However, despite this knowledge about NK cell-mediated immunomodulation and the effect of inflammatory mediators on NK cell function [27], the immunomodulatory activity of NK cells during antiviral mAb immunotherapies has been little studied. It is important to address this issue as inflammatory conditions resulting from viral infection and mAb therapy might modulate NK cell function.

We previously showed that NK cells are required for the optimal viral control of FrCasE retroviral infection upon treatment with the neutralizing 667 mAbs [39] through ADCC [19]. We also showed that the mAb-treatment of FrCasE-infected mice leads to increased frequencies of IFN-γ-secreting NK cells, suggesting that NK cells might participate in the modulation of the antiviral immune response upon mAb-treatment through the production of this proinflammatory cytokine. Here, we reported that the mAb treatment of infected mice preserves the functional activation of NK cells. Our work also shows that, in addition to being key cells in controlling viral spread, NK cells prevent immune dysfunction and have an essential role in inducing protective immunity upon mAb therapy. These findings highlight that it may be necessary to preserve the function and number of NK cells in order to obtain the protective immunity induced by mAbs. They also open new perspectives to improve mAb-based antiviral therapies through the development of therapeutic interventions aimed at harnessing the immunomodulatory properties NK cells. 

## 2. Materials and Methods

*Ethical statement*. Mice were bred and maintained under conventional, pathogen-free facilities at the Institut de Génétique Moléculaire de Montpellier. All experimental procedures were performed in accordance with the French national animal care guidelines (CEEA-LR-12146 approval).

*Viral stocks.* FrCasE viral stocks were produced, assayed and stored as described previously [8]. Briefly, the culture supernatants of *Mus dunni* embryo fibroblasts transfected with the FrCasE proviral clone [40] were used as viral stocks [41]. Viral titers were determined using a focal immunofluorescence assay (FIA) as previously described [42]. Dilutions of virus-containing samples were added to 25% confluent *Mus dunni* cell cultures in the presence of 8 µg/mL of polybrene. The cell-to-cell spread of replication-competent retroviruses was allowed to proceed for 2 days, and focus-forming units (FFUs) were visualized by the indirect immunofluorescence using the 667 mAbs [39] and a fluorescein isothiocyanate (FITC)-conjugated anti-mouse immunoglobulin.

*Viral infection, immunotherapy and mice follow-up*. Inbred 129/Sv/Ev mice (H-2D^b^ haplotype) were used in this study. Eight day-old 129/Sv/Ev mice were infected intraperitoneally (i.p.) with 50 µL of a virus suspension containing 50,000 FFUs and treated, or not, with 30 µg of 667 mAb 1 h post-infection (p.i.) and on days 2 and 5 p.i. by i.p. administration. Mice were examined at regular intervals for clinical signs of erythroleukemia, which was assayed by measuring the reduction in hematocrit, which is associated with anemia, and spleen swelling by the direct abdominal palpation on living animals or by direct examination after euthanasia [8,19]. They were euthanized when their hematocrits reached 35% (experimental endpoint) to prevent unnecessary pain. 

*Flow cytometry.* Spleen single-cell suspensions were obtained by the mechanical dissociation of the organs in phosphate-buffered saline (PBS). Bone marrow (BM) cell suspensions were obtained by dissection and the PBS-flushing of tibias and femurs. Cells were stained at 4 °C using fluorochrome-conjugated antibodies to: CD3e-FITC (145-2C11), CD4-BV450 (RM4-5), CD8-AAF (Ly2, 53-6.7), CD11b-AAF (M1/70), CD11c-BV450 (HL3), CD16/32-PeCy7 (2.4G2), CD19-PerCPCy5.5 or Pe-Cy7 (1D3), CD27- PerCPCy5.5 (LG3A10), CD45.2-BV500 (104), CD45R/B220- PerCPCy5.5 (RA3-6B2), Gr1-APC (RB6-8C5), Ly6G-PE (1A8), NKp46-BV450 (29A1.4), CD49b-PE (DX5), PD-1-FITC (RMP1-30), PD-L1-PE (MIH5), CD39-PeCy7 (24DMS1), CD69-PeCy7 (H1.2F3), IA/IE-FITC (2G9), IgM-PE (eB121-15F9), IgD-APC (11-26C), Ly49D-APC (4E5) (BD Bioscience, eBioscience or BioLegend). FrCasE-infected cells were assayed using an anti-Gag mAb (H34) [43] labelled with Alexa Fluor 647. Forward scatter area and forward scatter time-of-flight, as well as side scatter, were used to remove doublets from flow cytometry analyses. Cells were analyzed on a FACSCanto II flow cytometer (BD Bioscience) and the data were analyzed using the FlowJo software (Tree Star).

*ELISA of anti-FrCasE antibodies*. Mice were bled at the retro-orbital sinus to assay anti-FrCasE serum immunoglobulin concentrations. After clotting at room temperature for 15 min, blood samples were centrifuged at 8000× *g* for 10 min, and serum aliquots were stored at −20 °C until use. Plasma anti-FrCasE immunoglobulins were assayed by ELISA as already described [8]. Samples were diluted in PBS (0.15 M NaCl, 0.01 M Na phosphate, pH 7) containing 0.1% Tween 20 and 1% bovine serum albumin. A peroxidase-conjugated anti-mouse immunoglobulin G (IgG) (Serotec) was used as a secondary antibody.

*In vivo depletion of NK cells*. NK cells were depleted using the anti-asialo GM1 antibody (Wako Pure Chemical Industries, Ltd.). This depleting antibody was injected 1 day before infection and at days 1, 7, 13 and 19 p.i. (50 µL/injection). This antibody has been used to study the in vivo functions of NK cells in mouse strains lacking the NK1.1 allotype, which is a feature of 129 Sv/Ev mice [44].

*In vivo cytolysis activity*. Experiments were conducted as described in [8,19,45]. Briefly, red blood cell-free splenocytes were recovered from 10 day-old FrCasE-infected-, or non-infected, pups. Splenocytes from non-infected mice were labelled with the vital dye carboxy-fluorescein succinimidyl ester (CFSE; Molecular Probes) at a concentration of 0.5 μM (CFSE^low^ cells). Splenocytes from infected mice were labelled with 5 μM CFSE (CFSE^high^ cells) and pre-incubated, or not, with the 667 mAbs (the absence of 667 allows to quantify spontaneous cell death). Both cell populations were mixed at a 1:1 ratio before i.v. administration to recipient mice. Cytolysis activity against infected splenocytes was calculated from the ratio of CFSE^low^/CFSE^high^ cells in spleen assayed by flow cytometry 5 h later. 

*Flow cytometry assay of CD8^+^ T cells specific for FrCasE-infected cells*. Splenocytes were labelled with both an APC-conjugated anti-CD8^+^ T cell antibody and a PE-conjugated (major histocompatibility complex (MHC) class I H-2D^b^ tetramer (Beckman Coulter, Villepinte, France) displaying the immunodominant Friend virus GagL epitope [8,46] (D^b^-GagL tetramers) as previously described [8].

*NK cell, neutrophil and B cell isolation.* Single-cell suspensions of splenocytes were prepared from 8- to 12-week-old naive mice. NK cells (CD49b^+^CD3e**^-^**) were sorted (>98% pure) using a Becton Dickinson (BD) Biosciences FACSAria device. B cells were purified using anti-CD45R/B220 biotinylated antibody (Biolegend) and streptavidin magnetic beads (Miltenyi, Paris, France). Neutrophils were isolated from naïve mice bone marrow (BM). After the dissection of lower limbs, BM cell suspensions were collected by PBS-2% fetal bovine serum (FBS) EDTA (2 mM) flushing (25G needle) of tibias and femurs. BM cell suspensions were filtered with a 0.40 µm nylon strainer. Neutrophils were isolated using a magnetic-based cell-sorting (MACS) neutrophil isolation kit (>95% purity; Miltenyi Biotec, Paris, France). Cells were placed in culture in U bottom 96-well plates at a concentration of 1 million/mL in 10% FBS-containing RPMI medium.

*In vitro assessment of NK cell activation.* NK cells were cultured in 96-well plates at a density of 1.5 × 10^5^ cells/well in the presence, or in the absence, of IL-12 (5 ng/mL) and IL-18 (5 ng/mL) for 24 h. Activation was assessed by measuring the expression of a CD69 molecule by flow cytometry and by quantifying IFN-γ production. Soluble IFN-γ from cell-free supernatants of cultured NK cells was assayed using IFN-γ ELISA (eBiosciences, Paris, France). 

*In vitro co-culture experiments of NK cells and neutrophils.* NK cells activated or not with IL-12 (5 ng/mL) and IL-18 (5 ng/mL) were co-cultured with BM-purified neutrophils. Cells were co-cultured at a ratio of 1:1 (0.75 × 10^5^ NK cells with 0.75 × 10^5^ cells neutrophils) in 96-U well plates with 200 µL of medium per well for 24 h. Neutrophils cultured in the presence of IL-12 and IL-18 were used as controls. Granulocyte-colony stimulating factor (G-CSF) (R&D Systems, Noyal Châtillon sur Seiche, France) was added at a concentration of 10 ng/mL to neutrophil cultures to maintain cell viability. Neutrophil activation was assessed by measuring the increase in the CD11b molecule by flow cytometry and by measuring their BAFF release.

*BAFF protein release quantification.* Soluble BAFF (B-cell activating factor) from cell-free supernatants of cultured neutrophils was assayed using BAFF ELISA (enzyme-linked immunoabsorbent assay) (R&D Systems).

*In vitro co-culture of B cells and neutrophils.* A total of 1 × 10^5^ B cells were cultured with 1 × 10^5^ purified neutrophils in RPMI 1640 medium containing 10% FBS (vol/vol) with 10 ng/mL G-CSF (R&D) for 4 days at 37 °C, in the presence, or in the absence, of IFN-γ (100 ng/mL). After 4 days of co-culture, the activation of B cells was analyzed by evaluating the transition of IgD^+^IgM^−^ to IgD^+^IgM^+^ or IgD^+^IgM^+^ to IgD^−^ IgM^+^. 

*Statistical analyses.* Statistical analyses were performed using GraphPad Prism 9 (GraphPad Software, San Diego, US). Data were expressed as the means +/− SEM (standard error of the mean) and statistical significance was established using a parametric one-way ANOVA (analysis of variance) test with a Bonferroni correction for multiple comparisons or unpaired Student’s t tests when two groups were compared. *p* values lower than 0.05 were considered as statistically significant. 

## 3. Results

**Figure 1:**
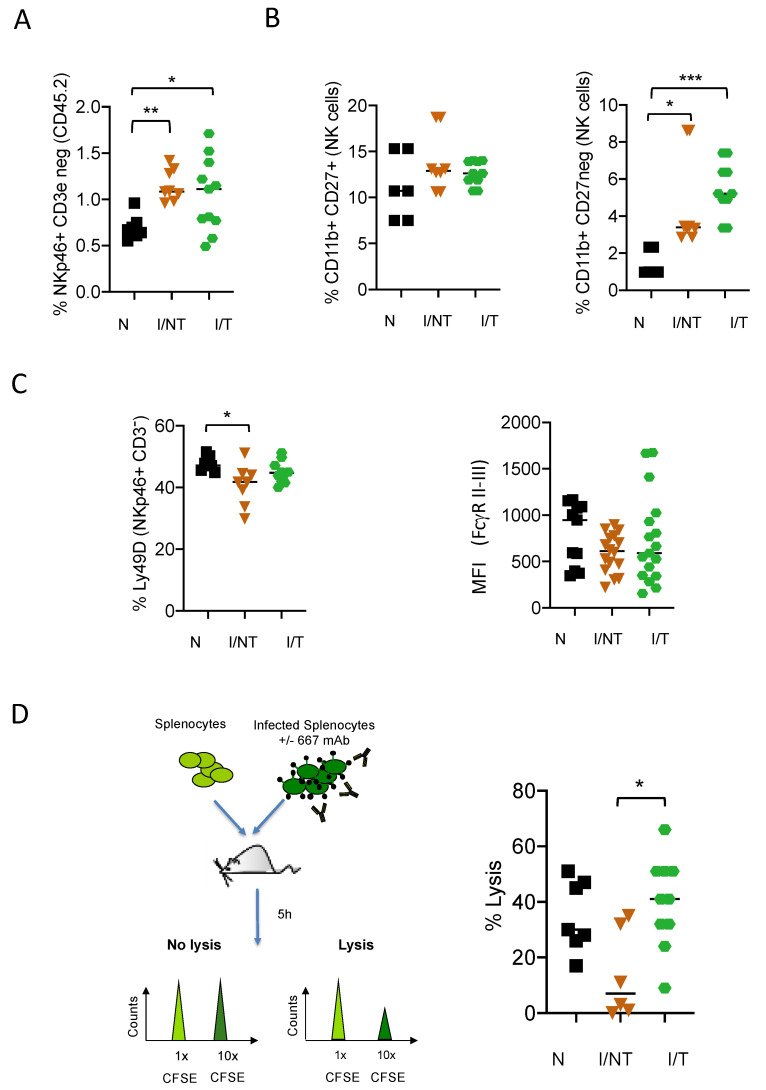
*Effect of monoclonal antibody (mAb) treatment of FrCasE-infected mice on NK cell recruitment and functional activation.* Eight-day-old pups were infected with the FrCasE retrovirus and treated or not with the 667 mAbs on days 0, 2 and 5 p.i.: (**A**) frequency of CD3^−^NKp46^+^ cells in the CD45.2^+^ leukocytic population at 14 days p.i.. The data presented correspond to 3 independent experiments with the following number of mice per group: naïve (*N*) (*n* = 7); infected/non-treated (I/NT) (*n* = 8); infected/treated (I/T) (*n* = 11); (**B**) maturation state of NK cells assessed by the expression of CD27 and CD11b in the CD3^-^NKp46^+^ population at 14 days p.i. The data presented correspond to 2 independent experiments with the following number of mice per group: naïve (*n* = 5); infected/non-treated (*n* = 8); infected/treated (*n* = 8); (**C**) assessment of the functional state of NK cells assessed by the expression of cell surface markers involved in the activation (Ly49D) and antibody-dependent cell-mediated cytotoxicity (ADCC) activity (FcγRs) at 14 days p.i. The data presented correspond to 3 independent experiments with the following number of mice per group: naïve (*n* = 7); infected/non-treated (*n* = 8); infected/treated (*n* = 10) for Ly49D and 4 independent experiments with the following number of mice per group: naïve (*n* = 11); infected/non-treated (*n* = 16); infected/treated (*n* = 17) for FcγRII/IIII; (**D**) ADCC activity of NK cells in naïve, infected/non-treated and infected/treated mice at day 30 p.i. Splenocytes from non-infected mice were labelled using 0.5 μM of the vital dye carboxy-fluorescein succinimidyl ester (CFSE) (CFSE^low^ cells; 1x and mixed at a 1:1 ratio with splenocytes from FrCasE-infected mice labelled using 5 μM CFSE (CFSE^high^ cells; 10x and pre-incubated, or not, with the 667 mAbs. Mixed cell populations were administered to the different groups of mice at day 30 p.i. Cytolysis was quantified 5 h later as described in Materials and Methods section. The data correspond to 3 independent experiments with the following number of mice per group: naive (*n* = 7); infected/non-treated (*n* = 6); Infected/treated (*n* = 15). (* *p* < 0.05; ** *p* < 0.01; *** *p* < 0.001).

*MAb treatment preserves the functional activation of NK cells.* As NK cells are key innate effector cells to control viral propagation upon mAb treatment, we first addressed NK mobilization and function in FrCasE-infected mice with, or without, 667 mAb treatment (infected/treated versus infected/non-treated). Passive immunotherapy was administered on the same day after the establishment of viral infection [8] and at days 2 and 5 post-infection (p.i.). Age-matched naïve mice were used as controls. NK cell recruitment was assessed in the spleen at day 14 p.i. To this end, we measured the frequency of CD3^−^NKp46^+^ cells in the spleens of mice from the different groups. The CD3^−^NKp46^+^ population mostly contains NK cells but may also include other innate lymphoid cells (ILC). We previously showed that over 95% of CD3^-^NKp46^+^ cells were CD49b^+^ (that distinguish NK cells from ILC), indicating that the vast majority of CD3^−^NKp46^+^ cells in the spleen display a NK phenotype [47]. As compared to naïve mice, NK cell abundance in both infected/treated mice and infected/non-treated animals was significantly higher (Figure 1A). However, NK cell abundance was comparable in both groups of infected mice with or without mAb treatment. We previously showed higher frequencies of IFN-γ-secreting NK cells in infected/treated mice as compared to infected/non-treated animals [19]. To further characterize the effect of mAb treatment on NK cell properties, we then assessed the maturation state of the recruited NK cells, by monitoring the cell surface expression of CD27 and CD11b molecules at day 14 p.i. (Figure 1B). Infected/non-treated as well as infected/treated mice showed similar frequencies of semi-mature (CD27^+^CD11b^+^) NK cells as compared to naïve mice. However, both groups of infected mice showed a higher frequency of fully mature (CD27^−^CD11b^+^) NK cells (notably infected/treated mice) (Figure 1B), showing an effect of viral infection and immunotherapy on NK maturation. We further assessed the functional state of NK cells by measuring the expression of other markers related to NK cell activation (i.e., Ly49D) and ADCC activity (FcγRs). Ly49D is an activating receptor that delivers stimulatory signals for target cell lysis. We observed a significant reduction in this activating receptor in NK cells from infected/non-treated mice as compared to naïve mice, suggesting that viral infection decreases the activation state of NK cells (Figure 1C). By contrast, no significant decrease in Ly49D expression was observed in the NK cells from infected/treated mice, suggesting an effect of mAb treatment on the preservation of NK cell function (Figure 1C). Regarding FcγRs, mouse NK cells are equipped with the activating receptor FcγRIII while they do not express the other murine FcγRs (i.e., FcγRI, FcγRIIB and FcγRIV) [48]. Neither viral infection nor mAb treatment modified the expression of FcγRIII (Figure 1C). Only background levels of FcγRI and FcγRIV were detected in NK cells, in agreement with published studies [48]. We then assessed the ADCC activity of NK cells in both infected/non-treated and infected/treated mice by using an *in vivo* cytotoxic assay performed at 30 days p.i. and treatment. Interestingly, we could observe that NK cells from infected/treated mice displayed a significantly higher ADCC activity than NK cells from infected/non-treated mice (Figure 1D). Overall, these results show an effect of mAb-treatment on the preservation of the functional activation of NK cells. 

**Figure 2:**
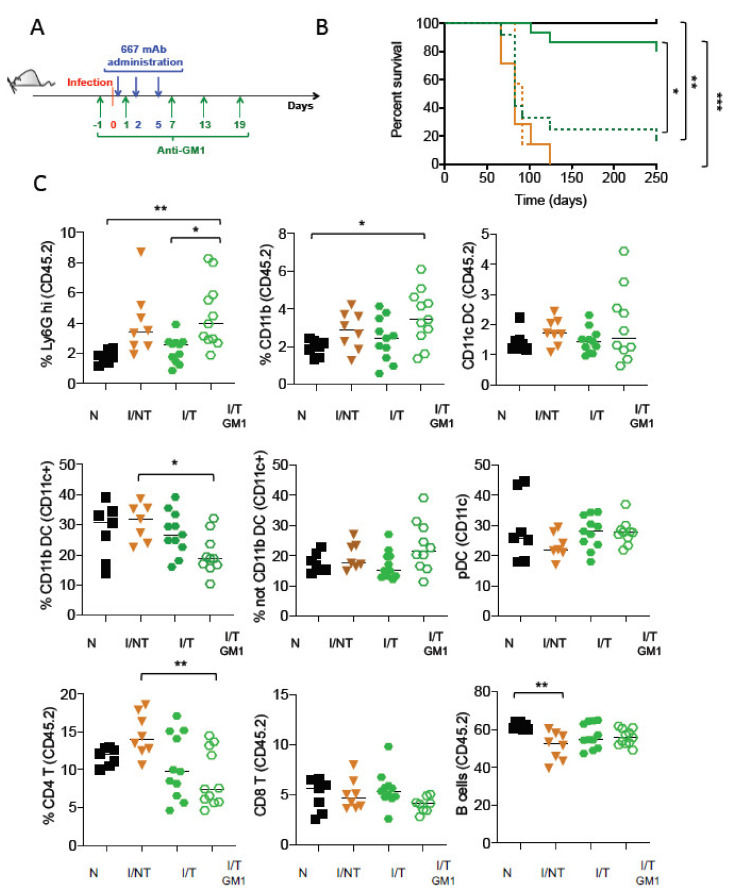
*The effect of NK cell depletion in disease progression and in the recruitment of immune cells upon FrCasE infection and mAb treatment*: (**A**) mice were infected with the FrCasE retrovirus and treated or not with the 667 mAbs as in Figure 1. Infected mice were treated as indicated with the anti-asialo-GM1 antibody to deplete NK cells. This depleting antibody was injected 1 day before infection and at days 1, 7, 13 and 19 p.i.; (**B**) effect of NK cell depletion on the survival of infected/non-treated and infected/treated mice. The data correspond to 2 independent experiments with the following number of mice per group: naïve (solid black line) (*n* = 6); naïve-GM1 (dotted black line, overlapping with solid black line) (*n* = 5), infected/non-treated (solid orange line) (*n* = 7); infected/non-treated-GM1 (dotted orange line) (*n* = 7); infected/treated (*n* = 15) (solid green line); infected/treated-GM1 (dotted green line) (*n* = 12); (**C**) frequency of immune cells in the spleen at days 14 p.i. measured in the CD45.2^+^ leukocytic population. The data correspond to at least 3 independent experiments with the following number of mice per group: naïve (*N*) (*n* = 7); infected/non-treated (I/INT) (*n* = 8); infected/treated (I/T) (*n* = 11), infected/treated-GM1 (I/T GM1) (*n* = 11, except for CD8 (*n* = 8) and DCs *n* = 10). (* *p* < 0.05; ** *p* < 0.01; *** *p* < 0.001).

*NK cell depletion in mAb-treated mice is associated with enhanced neutrophil recruitment and decreased frequencies of CD4^+^ T cells and CD11c^+^CD11b^+^ DCs at the infection site.* We previously showed that NK cells are crucial for the protection of infected/treated mice via efficient the control of viral propagation by 667-mediated ADCC [19]. Here, we assessed the consequences of NK cell depletion on the recruitment of different myeloid and lymphoid cells in the spleen (one of the main sites of viral propagation) at day 14 p.i. NK cells were depleted using an anti-asialo-GM1 antibody [44,49,50] (Figure 2A). This antibody led to the efficient depletion of NK cells (mostly semi-mature and mature NK cell subpopulations) in over 97% of mice (Supplementary Figure S1A). In addition, the administration of the anti-asialo-GM1 antibody to naïve mice did not significantly modify the splenic frequencies of different myeloid and lymphoid cells (except for a slight decrease in the frequency of CD8^+^ T-cells) (Supplementary Figure S1B), suggesting that NK cell depletion had little effect on homeostatic cell proliferation. No differences in the survival rate were detected in infected/non-treated mice in the presence, or in the absence, of NK cells (Figure 2B). Confirming our previous results, NK cells were key to achieving the long-term protection (>250 days p.i.) of infected/treated mice (Figure 2B) as its depletion drastically reduced mice survival despite mAb treatment. In the absence of NK-mediated viral control, we could observe a significantly increased recruitment of CD11b^+^ myeloid cells in the spleen of infected/treated mice, notably neutrophils (Figure 2C). This increased neutrophil frequency was associated with a higher percentage of spleen-infected cells (Supplementary Figure S2), as assessed by flow cytometry using the H34 antibody (recognizing a Gag protein epitope expressed on the surface of FrCasE-infected cells) [46]. NK cell depletion in infected/treated mice was also associated with a significant decrease in the frequencies of the CD11c^+^ CD11b^+^ DC subpopulation and CD4^+^ T cells, while hardly affecting the frequencies of other DC subpopulations or other lymphocytes subsets (i.e., CD8^+^ T cells and B cells) (Figure 2C). Thus, NK cell depletion in infected/treated mice leads to the modified splenic frequencies of innate and adaptive immune cells in the acute phase of infection.

**Figure 3:**
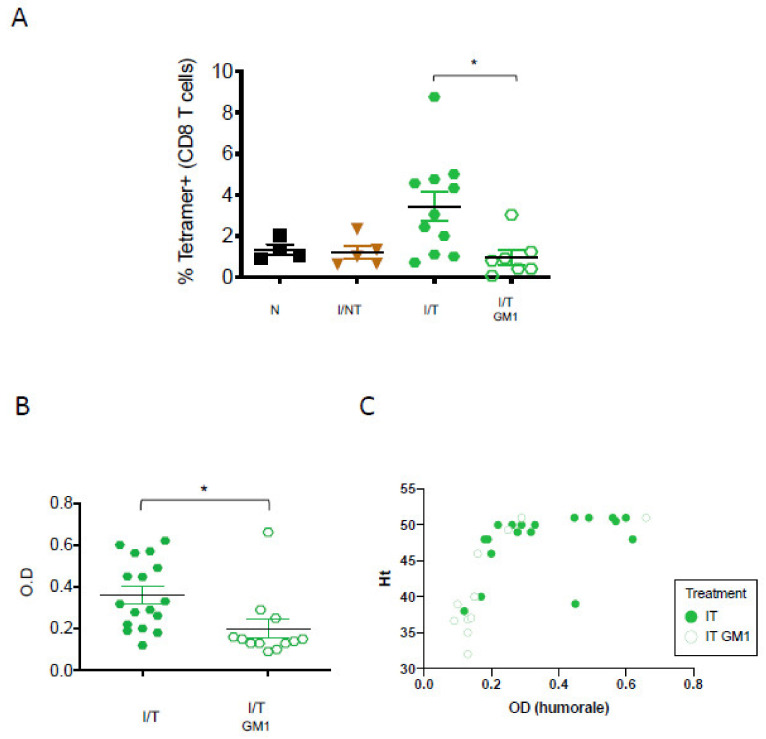
*Assay of FrCasE-specific CD8^+^ T cells and humoral responses in infected/treated mice in the presence and in the absence of NK cells.* NK cells were depleted in infected/treated mice as indicated in Figure 2A: (**A**) frequency of FrCasE-specific CD8^+^ T cells. Spleen cells were isolated at day 14 p.i. and the frequency of virus-specific CD8^+^ T cells in the total CD8^+^ T-cell population was assayed by flow cytometry using the H2D^b^-GagL MHC tetramer. The data correspond to 2 independent experiments with the following number of mice per group: naïve (N) (*n* = 4); infected/non-treated (I/T) (*n* = 5); infected/treated (I/IT) (*n* = 11), infected/treated-GM1 (I/IT GM1) (*n* = 7); (**B**) Seric FrCasE-specific IgG concentration was assayed by ELISA at 67 days p.i. The data represent 2 independent experiments with the following number of mice per group: infected/treated (*n* = 17), infected/treated-GM1 (*n* = 12). (* *p* < 0.05); (**C**) assessment of serum anti-FrCasE IgG levels in infected/treated (IT) and infected/treated, NK cell-depleted (IT-GM1) mice versus disease protection at day 67 p.i.

*NK cell depletion abrogates the induction of vaccinal effects by mAbs.* We showed that the mAb treatment of FrCasE-infected mice leads to enhanced virus-specific CD8^+^ T-cell responses as well as to protective humoral responses [8,19]. To identify the potential immunomodulatory functions of NK cells upon mAb treatment in addition to their cytotoxic activity, we then assessed the role of NK cells in the modulation of adaptive immune responses in infected/treated mice. We first addressed the cellular adaptive immunity by assaying the primary virus-specific CD8^+^ T-cell response in infected/treated mice at its peak (i.e., 14 days p.i.) [8] with or without NK cell depletion. We showed that the frequency of virus-specific CD8^+^ T cells in infected/treated mice was significantly reduced by NK cell depletion (Figure 3A). Interestingly, in contrast to the reduced splenic CD8^+^ T-cell frequencies observed in naïve mice upon NK cell depletion (Supplementary Figure S1B), NK cell-depleted, infected/treated mice did not show a decrease in the abundance of total CD8^+^ T cells (Figure 2C). This suggests that the impaired virus-specific CD8^+^ T-cell response observed in these mice is not the result of a bystander effect of NK cell depletion on homeostatic CD8^+^ T cells proliferation. This also argues in favor of an immunomodulatory role of NK cells in the enhancement of this arm of the cellular antiviral response upon mAb therapy.

Then, we addressed humoral immunity in infected/treated mice, depleted or not in NK cells. To this end, virus-specific IgG serum concentrations were assayed by ELISA at 67 days p.i. (i.e., peak of the humoral immune response observed in infected/treated mice) [19]. In addition, in the absence of NK cells, the serum concentration of anti-FrCasE IgG was significantly reduced (Figure 3B). The lower anti-FrCasE IgG serum levels observed in NK cell-depleted mice were globally associated with leukemia progression (i.e., decreased percentage of hematocrit) (Figure 3C), highlighting the contribution of a high humoral antiviral response in the protection against disease. These results show that NK cell depletion in infected/treated mice abrogate the induction of adaptive immune responses by the therapeutic mAb. 

**Figure 4:**
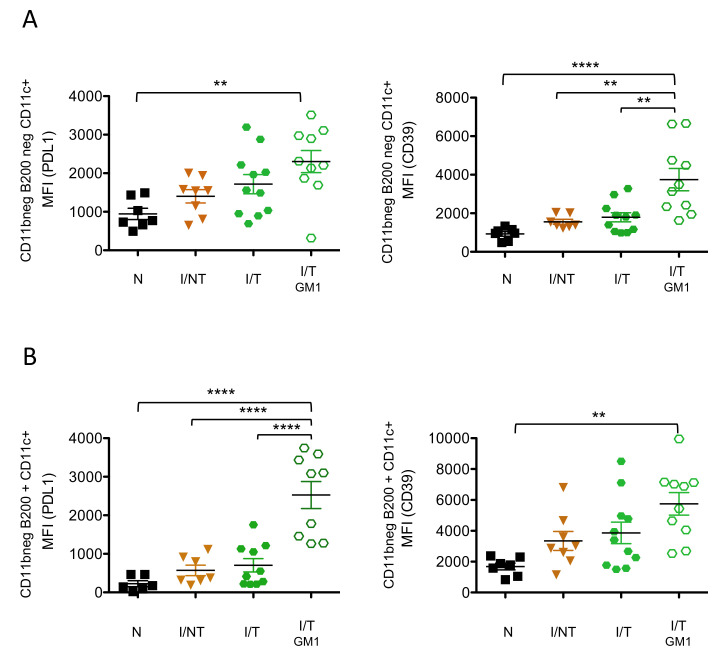
*Effects of NK cell depletion on the expression of inhibitory receptors on DCs*. NK cells in infected/treated mice were depleted, or not, as indicated in Figure 2A. Spleen cells were isolated at day 14 p.i. and flow cytometry analyzed to assess the expression levels of CD39 and PD-L1 on different subtypes of DCs: CD11c^+^CD11b^-^B220^-^ conventional DCs (**A**) and CD11c^+^CD11b^-^B220^+^ plasmacytoid DCs (pDCs) (**B**). The data correspond to 3 independent experiments with the following number of mice per group: naïve (N) (*n* = 7); infected/non-treated (I/NT) (*n* = 8); infected/treated (I/T) (*n* = 11), infected/treated-GM1 (I/T GM1) (*n* = 10) (** *p* < 0.01; **** *p* < 0.0001).

*NK cell depletion is associated with enhanced expression of molecules involved in immunosuppressive pathways on DCs and CD4^+^ T cells.* The lower humoral and virus-specific CD8^+^ T-cell responses observed upon NK cell depletion suggested that the absence of NK cells might lead to the development of impaired immune responses. This is all the more important to consider given that the absence of viral control by NK cells via ADCC mechanisms might contribute to immune dysfunction. Thus, we assessed the effect of NK cell depletion on the upregulation of cell surface molecules involved in immunosuppressive pathways in innate and adaptive immune cells. To this end, we measured the expression of inhibitory receptors such as programmed cell death 1 (PD-1), PD-ligand1 (PD-L1) and the CD39 on DCs and CD4^+^ T cells at 14 days p.i. (i.e., peak of the cellular primary immune response) in infected/non-treated mice as well as in infected/treated mice, depleted or not in NK cells. As compared to naïve mice, neither infected/non-treated nor infected/treated mice significantly upregulated the expression of CD39 and PD-L1 on DCs. On the contrary, NK cell depletion in infected/treated mice led to the upregulation of CD39 and PD-L1 on a subpopulation of myeloid DCs (CD11c^+^CD11b^−^) as well as on plasmacytoid DCs (pDCs) (Figure 4), raising the possibility that the absence of mAb-mediated viral control might contribute to the expression of these inhibitory receptors. However, the expression of CD39 on CD11c^+^CD11b^−^ DCs and the expression of PD-L1 on pDCs were significantly higher in NK cell-depleted, infected/treated mice as compared to infected/non-treated mice. Taking into consideration that both groups of mice display similar levels of viral propagation at 14 days p.i. [19], this suggests that the expression of the CD39 and PD-L1 immunosuppressive molecules on both subtypes of DCs upon NK cell depletion is not merely the consequence of the lack of viral control. We also assessed the effect of NK cell depletion on the expression of other molecules crucial for immunomodulation such us MHC-II. In contrast to the enhanced expression of immunosuppressive molecules in CD11c^+^CD11b^−^ DCs and pDCs, NK cell depletion did not significantly modify the expression of MHC-II on these types of DCs (Supplementary Figure S3). However, a significant decrease in MHC-II was observed in CD11c^+^CD11b^+^ DCs upon NK cell depletion in infected/treated mice. 

**Figure 5:**
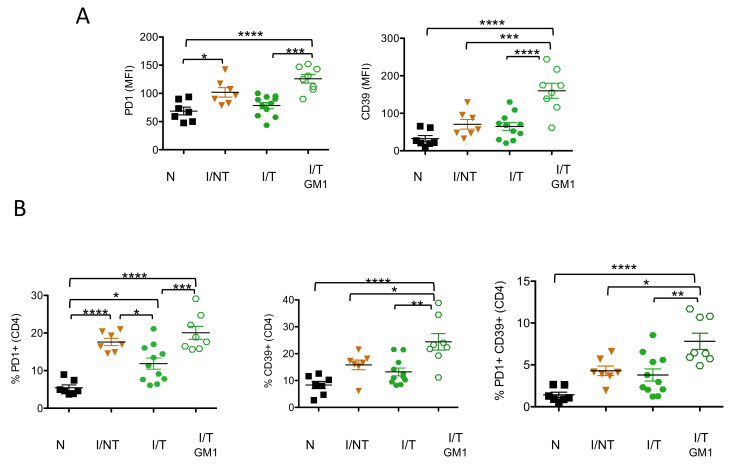
*Effects of NK cell depletion on the expression of inhibitory receptors on CD4^+^ T cells.* NK cells in infected/treated mice were depleted, or not, as indicated in Figure 2A. Spleen cells were isolated at day 14 p.i. and flow cytometry-analyzed to assess the expression levels of PD-1 and CD39 molecules on CD4^+^ T cells (**A**) and the frequency of PD-1- and CD39-expressing cells (**B**)*;* the data correspond to 3 independent experiments with the following number of mice per group: naïve (N) (*n* = 7); infected/non-treated (I/NT) (*n* = 6); infected/treated (I/T) (*n* = 11), infected/treated-GM1 (I/T GM1) (*n* = 8) (* *p* < 0.05; ** *p* < 0.01; *** *p* < 0.001, **** *p* < 0.0001).

Similar to CD11c^+^CD11b^-^ DCs, CD39 expression on CD4*^+^* T cells was only upregulated in NK cell-depleted, infected/treated mice, both in terms of median fluorescence intensity (MFI) and percentage of CD39-expressing cells, while no significant increased expression was observed in either infected/non-treated or in infected/treated mice as compared to naïve mice (Figure 5). In contrast, PD-1 expression on CD4^+^ T cells was increased in infected/non-treated mice but not in infected/treated mice, showing an effect of mAb treatment in preventing the upregulation of this inhibitory receptor. Similarly, the frequency of CD4^+^ PD-1-expressing cells was strongly increased in infected/non-treated mice and significantly reduced upon mAb treatment of infected mice. However, NK cell depletion in infected/treated mice led to a significant increase in both the expression of PD-1 and the frequency of CD4^+^ PD-1-expressing cells, reaching levels comparable to those observed in infected/non-treated mice. This suggests that the expression of PD-1 on CD4^+^ T cells might be associated with the lack of viral control. Finally, we assessed the percentage of CD4^+^ T cells expressing both inhibitory receptors. It is worthy of note that the depletion of NK cells in infected/treated mice resulted in a significantly enhanced percentage of double positive PD-1^+^CD39^+^ CD4^+^ T cells. Overall, our data show that the depletion of NK cells in infected/treated mice is associated with an increased expression of different inhibitory receptors on different DC subsets and on CD4^+^ T cells. This enhanced expression of molecules involved in immunosuppressive pathways might contribute to the induction of lower/inefficient adaptive immune responses.

**Figure 6:**
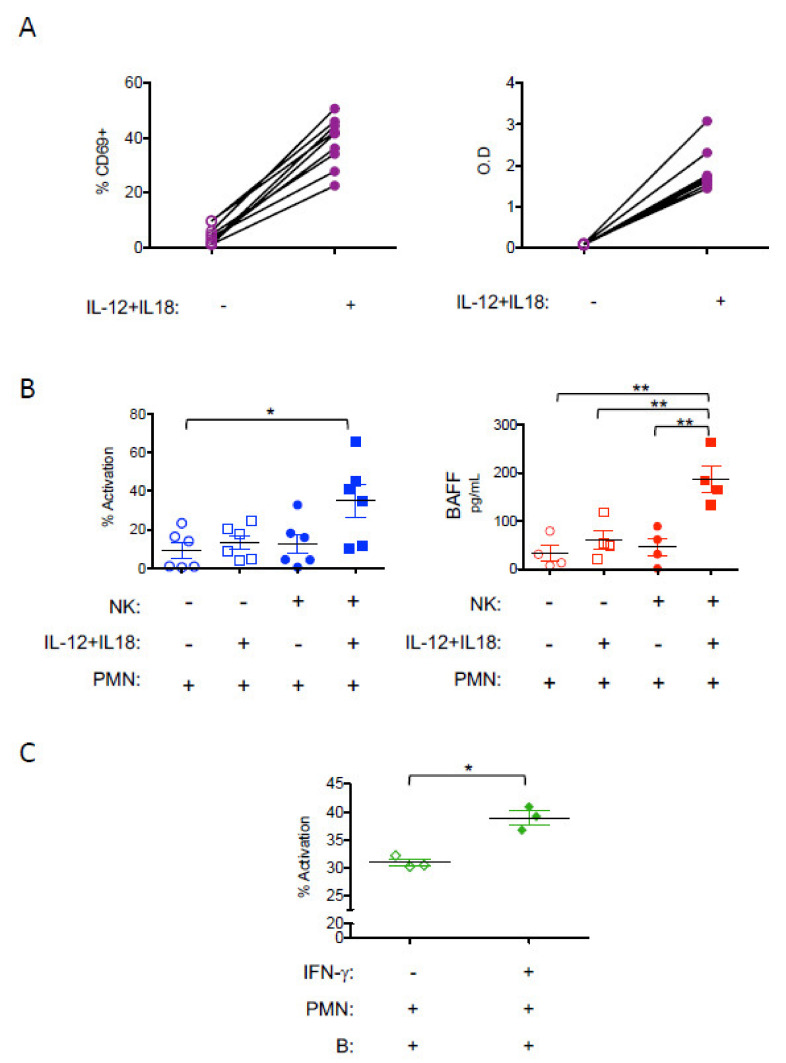
*Effect of NK-derived IFN-γ in the enhancement of the B-cell helper function of neutrophils.* NK cells from naïve mice were isolated and activated in vitro with IL-12 and IL18 and further used for co-culture experiments with neutrophils (PMN): (**A**) activation of NK cells by IL-12 and IL18, assessed by the upregulation of CD69 molecule (*n* = 8 independent experiments) and the secretion of IFN-γ (*n* = 9 independent experiments); (**B**) activation (increase in CD11b expression) and B-cell activating factor (BAFF) secretion capacity of neutrophils isolated from naïve mice co-cultured with IL-12 /IL18-activated NK cells (*n* = 6 for phenotype and *n* = 4 for BAFF secretion); (**C**) activation of B cells (transition of IgD^+^IgM^−^ to IgD^+^IgM^+^ or IgD+^+^IgM^+^ to IgD^−^ IgM^+^) isolated from naïve mice co-cultured with IFN-γ-activated neutrophils. The data correspond to 3 independent experiments (* *p* < 0.05; ** *p* < 0.01).

*NK-derived IFN-γ enhances the B-cell helper function of neutrophils.* The production of IFN-γ by NK cells has been shown to activate DCs and help T-cell responses [28,29]. However, whether and how NK-derived IFN-γ might play a role in enhancing B-cell responses has been much less studied. This is an important issue as the induction of potent antiviral humoral responses correlates with disease protection in infected/treated mice (Figure 3C) [19]. We, and others, have shown a role for IFN-γ in potentiating the B-cell helper function of neutrophils through increasing their BAFF release [19,51]. Based on our observations showing that the mAb-treatment of FrCasE-infected mice led to increased frequencies of IFN-γ-secreting NK cells in a neutrophil-dependent manner [19], we hypothesized that upon mAb-treatment, NK-derived IFN-γ could reciprocally affect neutrophils function. To address this issue, we assessed whether IFN-γ-producing NK cells were able to modulate neutrophil activation, notably their B-cell helper properties. To this end, we performed NK cell-neutrophil co-cultures. NK cells isolated from naïve mice were activated by IL-12 and IL-18, resulting in the enhanced expression of the CD69 cell surface activation marker and strong production of IFN-γ (Figure 6A). IL-12/IL-18-activated NK-cells led to the activation of neutrophils as observed by the increased expression of the CD11b cell surface marker (Figure 6B). Such activation of neutrophils was not observed by IL-12/IL-18 alone in the absence of NK cells. Moreover, IFN-γ-producing NK cells induced the secretion of BAFF by neutrophils (Figure 6B). Finally, IFN-γ-activated, BAFF-producing neutrophils led to the activation of B cells, as observed in neutrophil/B-cell co-culture experiments (Figure 6C). These results show that NK-derived IFN-γ induces BAFF secretion by neutrophils and enhances the B-cell helper function of the latter.

## 4. Discussion

The role of NK cells particularly in antibody-mediated immune protection has been associated with their capacity to eliminate infected cells via ADCC mechanisms. However, their role in the enhancement of adaptive antiviral immune responses upon mAb treatment has mostly been overlooked. Here, we show that NK cell depletion leads to reduced virus-specific CD8^+^ T-cells responses and humoral responses. Importantly, the lack of NK cells in mAb-treated mice is also associated with the enhanced expression of different molecules involved in immunosuppressive pathways in DCs and CD4^+^ T cells such as PD-1, PD-L1 et CD39. This suggests that fully functional NK cells are required to prevent immune inhibitory pathways. Furthermore, have also shown that in the absence of mAb-treatment, viral infection leads to the reduced cytotoxic activity of NK cells suggesting a role for mAb-mediated viral control in preventing the NK cells’ functional impairment. Such NK cell dysfunction has been observed in several acute and chronic viral infections [36,52,53,54,55]. Thus, NK functional status might have to be taken into consideration when initiating mAb-based immunotherapies as it might compromise the ADCC-mediated viral control by the mAb. This suggests that assessing/restoring the functional status of NK cells before antibody therapy might be key to achieving optimal therapeutic efficiency and preventing immune dysfunction.

We also showed that NK cell depletion in infected/treated mice leads to the modified frequencies of innate and adaptive immune cells at the infection site in the acute phase of infection. Notably, increased frequencies of effector myeloid cells such as neutrophils were observed, the latter being most probably recruited due to the lack of viral control. Whether or not these myeloid cells might acquire immunosuppressive properties and be involved in the induction of suppressive responses in a later phase of viral infection, as reported in other experimental models [56], will require further investigation. NK cell depletion is also associated with decreased frequencies of CD4^+^ T cells and CD11c^+^ CD11b^+^ DCs in infected/treated mice. It is worth noting that NK cell depletion in infected/treated mice also leads to a significant decrease in the expression of MHC-II in this DC subtype, which might contribute to the reduced immune responses observed in these mice. A limitation of our experimental model is the difficulty in discriminating between the bystander effect of NK cell depletion and the effect of viral infection on the frequency of splenic immune cells in infected/treated mice. This is due to the fact that both cell depletion and viral infection can affect the proportion and activation of cells. However, we were able to show that NK cell depletion in naïve mice did not significantly modify the frequencies of CD11c^+^ CD11b^+^ DCs and CD4^+^ T cells (Supplementary Figure S1B), suggesting that a potential bystander effect of NK cell depletion might not account for the decreased splenic frequencies of these cells in NK cell-depleted, infected/treated mice. 

Another important issue we addressed in this study is whether NK cell depletion affected not only the frequency but also the functional state of these myeloid cells. We found an increased expression of PD-L1 and CD39 inhibitory receptors on CD11c^+^ CD11b^-^ DCs and pDCs upon NK cell depletion in infected/treated mice. While PD-L1 expression has been considered as an activator marker in several studies, its expression on different subsets of DCs has been shown to compromise T-cell function [57,58]. Importantly, recent studies have shown that blocking PD-L1 on DCs improves DC function and leads to enhanced anticancer T cell immunity [57,58]. Furthermore, the expression of PD-L1 on pDCs has also been associated with the induction of tolerogenic immune responses in different experimental settings [59,60] as well as to bad prognosis in cancer patients [61]. This argues in favor of a role for the upregulation of PD-L1 on pDCs in the development of an impaired antiviral immune response that we observed in NK cell-depleted, infected/treated mice. Furthermore, higher PD-L1 expression on pDCs has been reported in non-treated-HIV infected patients as compared to those treated with antiretroviral therapy (ART), suggesting a role for the uncontrolled viral propagation on PD-L1 upregulation on these cells [62], in agreement with our findings.

Our data show an increased expression of PD-1 on CD4^+^ T cells from infected mice with uncontrolled viral propagation (i.e., infected/non-treated mice and NK cell-depleted, infected/treated mice). PD-1 is a negative regulator of T-cell proliferation but its expression is also upregulated in activated cells [63,64,65]. In the context of viral infections, the PD-1 upregulation on HIV-specific CD4^+^ T cells correlates with viremia [66] and has been shown to be important for regulating cytokine secretion [67,68,69,70]. In addition to inhibiting effector functions of different CD4^+^ T-cell subsets, PD-1 impairs HIV-specific T helper responses by limiting the expansion of these cells. Importantly, PD-1 blockade on CD4^+^ T cells has been shown to restore HIV-specific CD4^+^ T-cell function (by increasing their cytokine production) that in turn enhances cytokine secretion by NK cells [67,68,69]. Whether the increased PD-1 expression in our experimental model accounts for an activated status of CD4^+^ T cells or whether it mediates immune suppression will require further investigation. Nevertheless, our data suggest that the expression of PD-1 on CD4^+^ T cells might be associated with the lack of viral control and could be potentially involved in the induction of impaired CD4 T-cells responses, notably if it is co-expressed with other inhibitory receptors (i.e., CD39) as observed in NK cell-depleted, infected/treated mice (Figure 5). In keeping with this, PD-1 blockade recovered the activity of exhausted PD-1^hi^CD39^+^ CD4^+^ T cells [71].

The CD39/adenosine pathway is involved in immunosuppressive responses in different pathological contexts, including chronic viral infection [72,73]. However, most of the studies have reported the increased expression of CD39 on T cells [74,75] while its upregulation on DCs has been much less studied. It is worth noting that CD39 expression by tumor-infiltrating DCs has been shown to be involved in T cells inhibition [76] as well as in attenuating inflammation and autoimmune responses [77,78] but its upregulation has not been reported in the context of viral infections. Our data showing the enhanced expression of CD39 on CD4^+^ T cells corroborate previous reports and are in agreement with immunosuppressive responses observed in viral infected individuals [74]. Furthermore, the upregulation of CD39 on DCs observed in NK cell-depleted, infected/treated mice provides new insights into the modulation of this inhibitory receptor expression in the context of viral infections and suggests a role for the lack of NK cells in this process. Overall, our findings suggest that the increased expression of molecules involved in immunosuppressive pathways on DCs and CD4^+^ T cells observed in NK cell-depleted, infected/treated mice might contribute to the abrogation of vaccinal effects observed in these mice together with other cellular and molecular mechanisms involved in immune dysfunction. 

The immunomodulatory role of NK cells and their cross-talk with multiple cells of the adaptive and innate immune system have been shown to be mediated by several cellular molecular mechanisms [23,24,25]. While the role of NK-derived IFN-γ production in the enhancement of T-cell immune responses has been extensively described [28,29,79], much less is known about the role of NK-derived IFN-γ in enhancing B-cell responses. Similarly, the cross-talk between NK cells and neutrophils and their potential cooperation in modulating antiviral immune responses is still ill-understood. In this respect, NK cells have also been shown to shape neutrophil function and survival [30,32,80]. However, thus far, the neutrophil activation by NK cells has mostly been assessed by measuring their expression of cell surface markers, reactive oxygen species (ROS) production and the secretion of several proinflammatory cytokines. Our work describes an underappreciated mechanism of NK cell–neutrophil cross-talk and suggests a potential cooperation between NK cells and neutrophils in improving antiviral immune responses. Importantly, our findings provide new insights into the role of IFN-γ-secreting NK cells in potentiating B-cell responses by enhancing the BAFF secretion capacity of neutrophils. Consistently, they also show a role for IFN-γ-activated neutrophils on B-cell activation. These results are in an agreement with the B-cell helper role of neutrophils in mAb-treated infected mice that we previously reported and which was associated with increased frequencies of IFN-γ-secreting NK cells. Moreover, taking into consideration that BAFF production by neutrophils contributes to the enhancement of humoral responses, including in the context of viral infections and antiviral mAb-therapy [19,51,81,82], our work suggests that the effect of NK cells in modulating the antiviral humoral response upon mAb therapy might be in part mediated by enhancing the B-cell helper function of neutrophils via IFN-γ production. However, other mechanisms might also be involved, such as the enhancement of T-cell helper CD4^+^ responses as a consequence of NK–DC cross-talk, as previously reported [28,29,31]. Addressing this issue will require further investigation. 

Another potential limitation of our study is the administration of the anti-asialo-GM1 antibody which is less specific than other NK cell-depleting antibodies (i.e., anti NK1.1. antibody). This antibody was used due to the genetic background of the mice used in this study (129/Sv/Ev) which do not express the NK1.1 allotype [44]. Although the anti-asialo-GM1 antibody has been shown to deplete basophils in different mouse strains (including C57BL/6, BALB/c, C3H, and A/J mice) [83], it is generally used in strains lacking the NK1.1. [44,49,50]. We cannot rule out a potential role for basophils in the dysfunction of the immune response observed in infected/treated mice upon anti-asialo-GM1 administration. However, it has been shown in different experimental settings that basophil deficiency does not impair humoral and cellular immune responses and that these cells are dispensable for inducing protective immunity [84,85]. Although this issue will require further investigation, these observations suggest that a bystander effect of basophil depletion is not likely to be involved in the abrogation of the vaccinal effects observed upon anti-asialo-GM1 antibody administration in infected/treated mice. In addition, arguing in favor of a key role for NK cells in the modulation of the antiviral immune response in infected/treated mice, NK cells have also been shown to be involved in the induction of vaccinal effects by therapeutic mAbs in the context of cancer pathologies, in agreement with our observations. Notably, the role of NK cells in DC activation and the enhancement of T-cell responses has been reported upon mAb-treatment in a cancer mouse model [86] as well as in clinical studies including head and neck cancer patients. [87]. However, whether and how mAb treatment led to the modulation of the humoral response in a NK-dependent manner was not assessed. 

Our work showing that NK cells are key immune actors in the induction of vaccinal effects by antiviral mAbs might have therapeutic implications. Thus, if the ADCC-mediated capacity of NK cells is impaired as a consequence of the ongoing viral infection, it will be essential to restore NK cell function prior to antibody therapy to achieve the optimal therapeutic effect and prevent immune dysfunction. Furthermore, taking into account the enhanced expression of inhibitory receptors on different immune cells (i.e., PD-1, PD-L1, CD39) observed in the absence of viral control due to NK cell depletion, mAb-treatments might be associated with mAbs targeting these immune checkpoints [57,71,88,89]. Finally, other complementary approaches, such as the use of engineered mAbs [90,91] or antibody-derived molecules (i.e., bi-specific antibodies) [92], to enhance FcγR-triggering on NK cells, might not only allow superior ADCC, but also enhance the IFN-γ-secretion capacity of NK cells eventually leading to enhanced cellular and humoral immune responses. Our work opens new perspectives to improve the therapeutic efficiency of antiviral mAb through the development of novel therapeutic interventions aimed at harnessing the immunomodulatory properties of NK cells [33,34,35,36]. 

## 5. Conclusions

Our work identified new cellular and molecular mechanisms involved in the induction of antiviral protective immunity by mAbs and highlighted a key immunomodulatory role of NK cells in this effect. We show that antiviral mAb therapy preserves NK cell function. Furthermore, we report a key role of NK cells in both preventing immune cell dysfunction (i.e., the upregulation of immunosuppressive molecules on DCs and CD4^+^ T cells) as well as in inducing potent antiviral humoral and cellular responses. Finally, our observations also suggest a potential cooperation between NK cells and neutrophils in improving antiviral humoral responses during antibody therapy. This occurs through the enhancement of the B-cell helper function of neutrophils by NK cell-derived-IFN-γ production. Overall, our findings might guide the design of improved mAbs-based immunotherapies.

## Data Availability

All data is contained within the article and supplementary material.

## Supplementary Materials

The following are available online at https://www.mdpi.com/2076-393X/9/2/137/s1. Figure S1: (**A**) Efficacy of NK depletion by the anti-asialo-GM1 antibody in naïve and infected/treated mice. (**B**) Effect of NK cell depletion in the frequency of splenic immune cells in naïve mice. Figure S2: Correlation between frequency of splenic neutrophils isolated from FrCasE-infected mice and frequency of FrCasE infected cells at day 14 p.i. Figure S3: Effects of NK cell depletion on the expression of MHC-II molecule on DC.

## Abbreviations

ADCC, antibody-dependent cell cytotoxicity; ADCP, antibody-dependent cellular phagocytosis; ART, antiretroviral therapy; BAFF, B-cell activating factor; BM, bone marrow; CCL, chemokine (C-C motif) ligand; CD, cluster of differentiation; CDC, complement-dependent cytotoxicity; CFSE, carboxyfluorescein succinimidyl ester; DCs, dendritic cells; Fab, fragment of antigen binding; FBS, fetal bovine serum; Fc, fragment crystallizable; FcR, Fc receptor; FFU, focus-forming unit; FIA, focal immunofluorescence assay; G-CSF, granulocyte-colony stimulating factor; GM-CSF, granulocyte-macrophage colony-stimulating factor; HIV, human immunodeficiency virus; ICs, immune complexes; IFNγ, interferon γ; IgG, immunoglobulin G; IL, interleukin; ILC, innate lymphoid cells; I/NT, infected non-treated; I/T, infected-treated; mAbs, monoclonal antibodies; MHC: Major histocompatibility complex; N, naïve; NK, natural killer; PBS, phosphate buffer saline; PD1, programmed cell death 1; pDC, plasmacytoid DC; PDL1, programmed death-ligand 1; p.i., post-infection; SEM, standard error of the mean; ROS: reactive oxygen species;TNFα, tumor necrosis factor.
