# Supplementary material for: Immunomodulatory Role of NK Cells during Antiviral Antibody Therapy
Source: Vaccines (Basel). 2021 Feb 8;9(2):137. doi: 10.3390/vaccines9020137 (PMC7914599; doi:10.3390/vaccines9020137)
Supplement: Supplementary file 1 [file vaccines-09-00137-s001.pdf]

## Supplementary Figures and Figure Legends

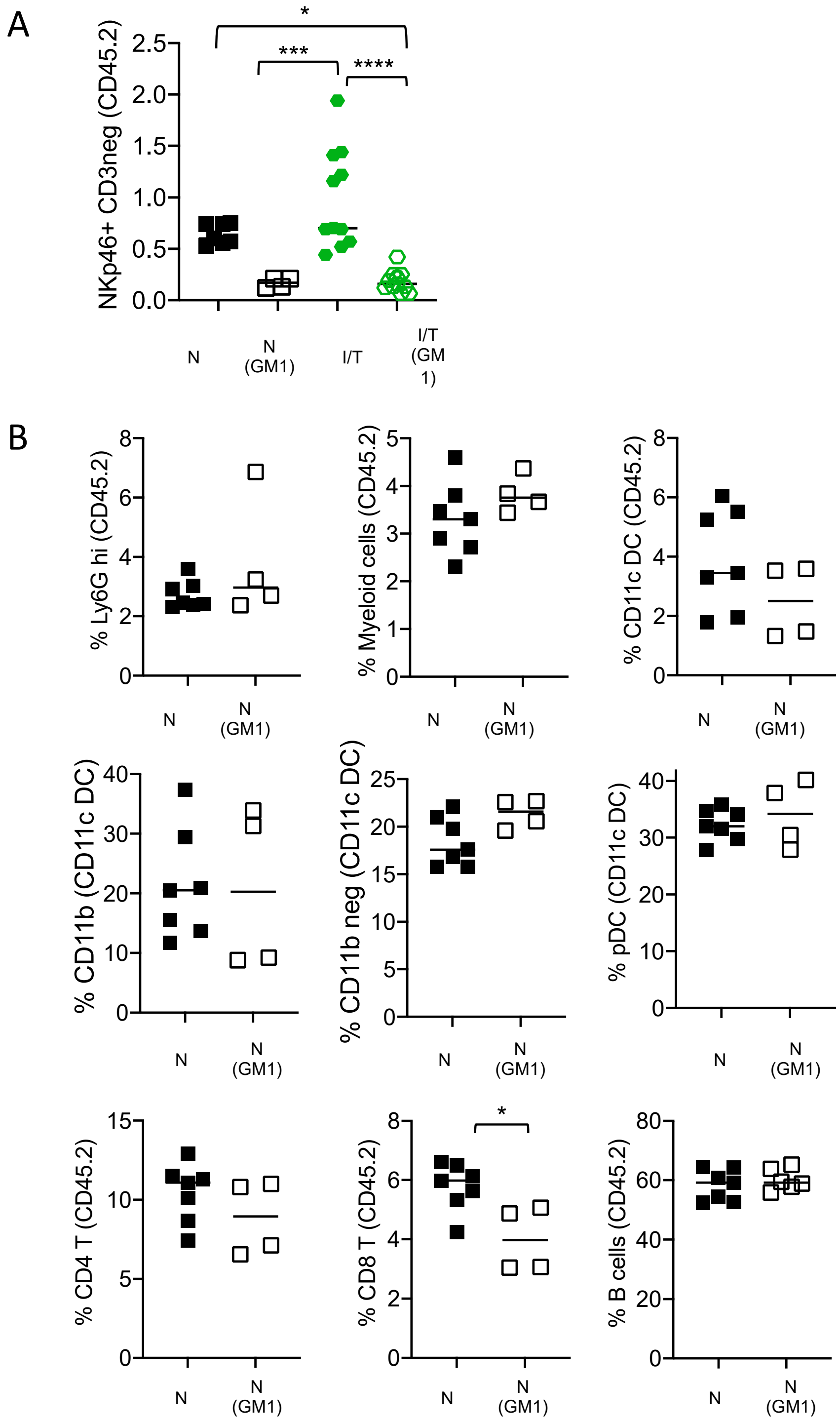


**Supplementary Figure 1.** (**A**) *Efficacy of NK depletion by the anti-asialo-GM1 antibody in naïve and infected/treated mice.* Mice were infected with the FrCasE retrovirus and treated with the 667 mAb as in Figure 1. The anti-asialo-GM1 antibody was injected 1 day before infection and at days 1, 7, 13 and 19 p.i. Naïve mice received equivalent anti-asialo-GM1 antibody treatment (same number of doses and same quantity of depleting antibody per dose). Efficiency of NK cell depletion was 1 day after the last anti-asialo-GM1 antibody administration (i.e., at 14 days p.i.). The data correspond to 3 independent experiments with the

following number of mice per group: naïve (*N*) (*n*=7); naïve-GM1 (N-GM1)(*n*=4); infected/treated (I/T) (*n*=11), infected/treated-GM1 (I/T GM1) (*n*=11); (**B**) *effect of NK cell depletion in the frequency of splenic immune cells in naïve mice.* Frequency of immune cells in the spleen at 1 day after anti-asialo-GM1 antibody administration was measured in the CD45.2+ leukocytic population. The data correspond to at least 3 independent experiments with the following number of mice per group: naïve (*N*) (*n*=7); naïve GM-1 (*n*=14) (**p* < 0.05; ****p* < 0.001, *****p* < 0.0001).

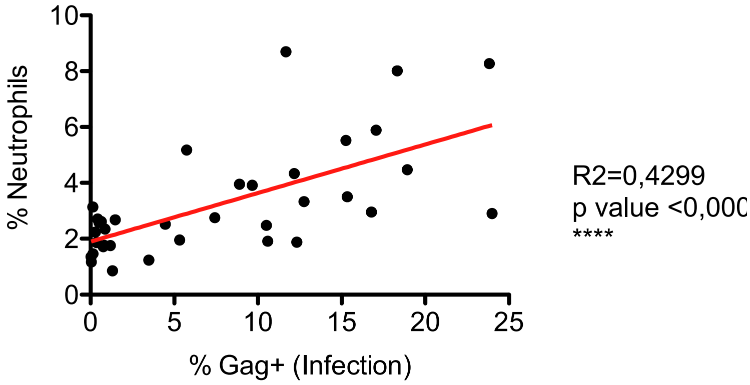


**Supplementary Figure 2.** *Correlation between the frequency of splenic neutrophils isolated from FrCasE-infected mice and the frequency of FrCasE infected cells at day 14 p.i.* Correlation was analyzed using the Pearson correlation test. The data correspond to 3 independent experiments (*****p* < 0.0001).

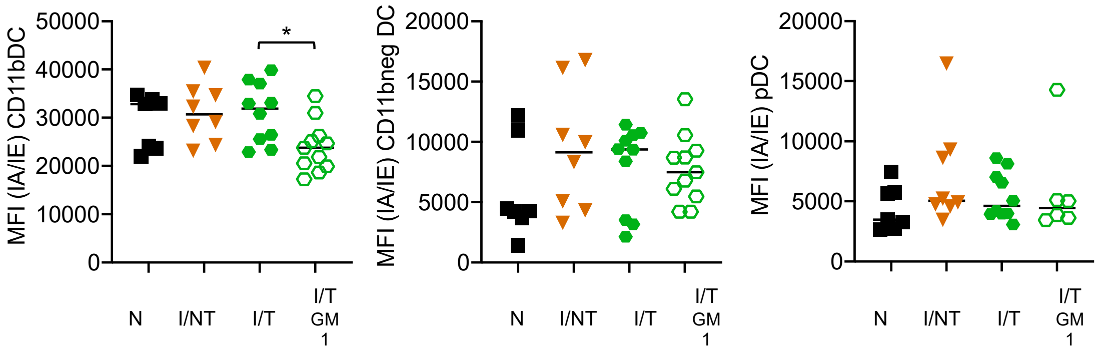


**Supplementary Figure 3.** *Effects of NK cell depletion on the expression of the MHC-II molecule (IA/IE) on DCs***.** NK cells in infected/treated mice were depleted, or not, as indicated in Figure 2A. Spleen cells were isolated at day 14 p.i. and flow cytometry-analyzed to assess the expression levels of MHC-II on different subtypes of DC. The data correspond to 3 indepen
dent experiments (**p* < 0.05).
